# Heart Failure in South America

**DOI:** 10.2174/1573403X11309020007

**Published:** 2013-05

**Authors:** Edimar Alcides Bocchi

**Affiliations:** Heart Institute (InCor), São Paulo University Medical School, Brazil

**Keywords:** Heart failure, cardiomyopathy, rheumatic fever, Chagas’ disease, epidemiology, valve disease, rheumatic fever, endomyocardial fibrosis, hypertrophic cardiomyopathy, restrictive cardiomyopathy, treatment, prognosis.

## Abstract

Continued assessment of temporal trends in mortality and epidemiology of specific heart failure in South America is needed to provide a scientific basis for rational allocation of the limited health care resources, and strategies to reduce risk and predict the future burden of heart failure. The epidemiology of heart failure in South America was reviewed. Heart failure is the main cause of hospitalization based on available data from approximately 50% of the South American population. The main etiologies of heart failure are ischemic, idiopathic dilated cardiomyopathy, valvular, hypertensive and chagasic etiologies. In endemic areas, Chagas heart disease may be responsible by 41% of the HF cases. Also, heart failure presents high mortality especially in patients with Chagas etiology. Heart failure and etiologies associated with heart failure may be responsible for 6.3% of causes of deaths. Rheumatic fever is the leading cause of valvular heart disease. However, a tendency to reduction of HF mortality due to Chagas heart disease from 1985 to 2006, and reduction in mortality due to HF from 1999 to 2005 were observed in selected states in Brazil. The findings have important public health implications because the allocation of health care resources, and strategies to reduce risk of heart failure should also consider the control of neglected Chagas disease and rheumatic fever in South American countries.

## INTRODUCTION

Major differences in populations were reported concerning cardiovascular event rates [1]. In developed countries reduction in cardiovascular (CVD) mortality has been reported over the last decades, however, and in some eastern European countries, mortality from CVD remains exceedingly high [2-3]. Chronic CVD have been considered a public health only in developed countries and among the elderly [4]. 

Heart failure, a chronic CVD, is considered as endemic in developed countries [5]. Heart failure is the most common cause of hospitalization in patients older than 65 years in the United States [6]. However, the overall rates of risk-adjusted hospitalization for HF declined 30% from 2845 per 100 000 person-years in 1998 to 2007 per 100 000 person-years in 2008 [7]. Authors have been attributed this decline in hospitalizations to reductions in the incidence of coronary artery disease, improved control of blood pressure, increased use of evidence-based therapies, and possibly changes in admission thresholds. In contrast to this significant reduction in hospitalizations for HF, 1-year risk-adjusted all-cause mortality rate declined minimally but remained high at approximately 30%.

However, published data reporting prevalence, prognosis, hospitalization, and death from HF in under-developed countries is scarce. 

## OBJECTIVE

This review was undertaken to examine trends in cardiovascular mortality and all causes in Brazil, the largest country in South America, and to focus on the burden from HF in South America. 

## SEARCH STRATEGY

A comprehensive literature search was performed using electronic bibliographic databases (e.g. CLINICALTRIALS.GOV, PubMed, COCHRANE, Elsevier/Science Direct, SciELO, LILACS) using the following keywords: heart failure, epidemiology and mortality. The search was limited to articles and trials with data from South America. The review was based on selected articles between 1998 and 2012. Reference lists from selected articles and reviews were also examined for further relevant articles, and we have cited those with information about epidemiology. Mortality ratios were derived from the Brazilian Ministry of Health data available electronically from 1983 to 2003 [8]. Also, data concerning HF as a cause of death in 2006 in the São Paulo State (Brazil) were obtained from the SEADE Foundation (Fundação Sistema Estadual de Análise de Dados) [9].

## CARDIOVASCULAR MORTALITY

The leading cause of death in Brazil was disease of the circulatory system [10]. The most important cause of cardiovascular death was cerebrovascular death followed by ischemic heart disease, other heart disease, acute myocardial infarction, hypertensive disease, atherosclerosis, and rheumatic disease [6]**. **Published data have demonstrated increase in mortality from ischemic heart disease, acute myocardial infarction, and hypertensive disease. Reduction in mortality was observed for cerebrovascular death, other heart diseases, atherosclerosis and rheumatic disease [6]. 

## HEART FAILURE

The lifetime risk of HF places its epidemic in perspective as a public health issue [11]. Most of the knowledge about HF is derived from North American and European studies. In contrast, in developing world no population-based study has been reported, and scarce information comes from data gathered in clinical trials or hospital-based studies [12]. Data obtained from SEADE Foundation concerning mortality of São Paulo State (Brazil) with estimated 41 654 020 habitants and limited to year 2006, demonstrated that HF or etiologies associated with HF except primary valvular disease are responsible by 6.3% of total deaths [7]. Thus, the real magnitude of the HF burden in South America is partially known (Table **1**). However, the increment of life expectancy in developing countries presages a rise in the incidence and prevalence of HF globally [13].

The mortality reduction related to improvement in prevention and treatment of acute coronary syndromes, coupled with an incremental of overall life expectancy has led to a relative enhancement of HF prevalence, especially from ischemic cardiomyopathy [14]. In developing countries the ischemic etiology also predominates, but, although to a lesser amount. Other etiologies such as rheumatic (Africa, Asia, Oceania and Latin America), Chagasic (Latin America) and hypertensive (African and African Americans) are also prominent. (Table **1**) In Brazil and all South America the epidemiologic data about HF are scarce and mostly related to populations from reference centers comprising inpatients and outpatients under ambulatory care [9]. Also, the relative prevalence of systolic HF and HF with preserved systolic left ventricular is not well documented. In a hospitalized elderly HF Argentinean small population, HF with preserved systolic function was more frequent especially in women [15].

A limited study including mortality rates due to HF obtained of three states of Brazil from 1999 to 2005, showed a decrease trend in mortality rates, except in the group aged 80 years or older [16]. 

## STUDIES OF HEART FAILURE INPATIENTS

The most robust Brazilian population information comes from the Ministry of Health (DATASUS) and is related to the number of hospitalizations because of HF [6]. Overall, cardiovascular diseases are the third most frequent cause of hospitalization. From 743763 hospitalizations due to cardiovascular diseases in 2007, 39.4% were associated to HF (70% of the cases in the age group above 60 years) [6]. The mortality ranged was from 6.58 to 6.95% and the mean hospitalized period was days was 5.8 days. Published data from DATASUS comparing hospitalizations between 1992-1993 and 2008-2009 showed a 32% reduction in hospitalizations for heart failure, with increase of 15% in mortality and of 25% in days of hospitalization [17]. 

In a Brazilian report including 903 hospitalized patients from a tertiary center with diagnosis of HF, the medium age was 52 years, with the ischemic etiology being the most frequent (33%), followed by the idiopathic (26%), valvular (22%), hypertensive (7%) and chagasic etiology(6%) [18]. However, in a general hospital of Antigua [19], reporting on a population of 293 patients with HF as the diagnosis of discharge, hypertensive etiology was the most prevalent etiology (41%), followed by the ischemic etiology (33%).

An analysis of 5 Argentinean registries with decompensated HF over the last decade showed that only in the first study (from 1993) there was a higher prevalence of the hypertensive etiology in relation to the ischemic (32.3% versus 28.7%) [20]. In the other 4 studies the ischemic (range from 27.4% to 38.4%) was more frequent than hypertensive etiology (from 18.2% to 23.7%). In those studies the medium age was above 60 years, with a high frequency of valvular etiology (range from 16.4% to 21.7%), and a low frequency of the chagasic etiology (range from 1.3% to 8.4%).The presence of preserved systolic function in this population was reduced (variation form 20% to 36%). 

The Brazilian EPICA-Niterói study compared decompensated HF populations from public versus private tertiary hospitals, showing medium age of 61 years and 66% of ischemic etiology at public hospitals, and respectively 72 years with 62% of ischemic at the private services [21]. In another Brazilian study of 212 decompensated HF patients from an emergency department of a tertiary hospital, a mean age of 60 years was shown, with high prevalence of the ischemic etiology (30%), followed by hypertensive (21%), valvular (15%) and Chagasic (15%) etiologies, and 45% of patients with preserved systolic function [22].

In the BELIEF multicenter study (Brazilian Evaluation of Levosimendan Infusion Efficacy) of 182 decompensated HF recently published by Bocchi *et al.*, reported younger (55 years) and more critical patients, since the required inclusion criteria the need for vasoactive drugs [23]. The HF etiology was ischemic in 34,1%, chagasic 21,4% and hypertensive in 13,2% of patients. The race was Caucasian in 58.2% and Afro-Brazilian in 36.8% [24].

A comparison of decompensated HF patients between tertiary hospitals in Brazil and United States showed that U.S. patients were older, had higher prevalence of ischemic etiology and less previous hospitalization for congestive HF than Brazilian patients, but similar comorbidities and left ventricular function [25]. Length-of-stay was shorter and in-hospital mortality was lower in the U.S. cohort, but fewer clinical events within 3 months after discharge were observed in Brazilian patients. In order to improve HF management, potential factors that might be associated with differences need to be evaluated in future studies. As an example, prescription of angiotensin-converting enzyme inhibitors at discharge from hospital was lower in the U.S. hospital but β-blockers prescription was higher in Brazilian patients.

Hospital readmission and emergency necessity was reported up to 51.2 % during a year of follow-up, with a rate of re-hospitalization of from 36% to 25.8% [26]. Hospitalization for HF presented slight variations with higher peak admission during the fall and winter [27].

In a Brazilian cohort, clinical predictors of HF with preserved left ventricular ejection fraction as a cause for hospitalization were age > 70 years old, female gender, non-ischemic etiology, atrial fibrillation or flutter, anemia, pulse pressure > 45 mmHg, and absence of EKG abnormalities [28].

Also, it was reported that history of HF was predictor in addition to other factors of higher in-hospital mortality in hospitalized older patients admitted to a geriatric care unit by non HF cause [29]. 

## STUDIES OF HEART FAILURE OUTPATIENTS

In the 1990’s, a Brazilian cohort study reported on 1220 outpatients followed in a specialized HF clinic, showing a mean age of 45 years. The idiopathic etiology was present in 37%, followed by chagasic (20%), ischemic (17%) and hypertensive in 14% of patients [30]. Of note, the chagasic etiology was associated with the worst prognosis [31]. In contrast, in the Argentinean GESICA study 40% of the patients had previous myocardial infarction, the idiopathic etiology was present in 20% and the chagasic in 10% [32]. In Mexico, from a population sample of 45 patients with systolic HF accompanied at a HF clinic of a tertiary hospital, 47% of patients had ischemic etiology and 44% idiopathic etiology, with a mean age of 61 years [33]. 

In the Brazilian REMADHE clinical trial of systolic heart failure patients followed at a tertiary hospital, Bocchi *et al.* described a higher prevalence of ischemic etiology (22%-28%). The chagasic etiology was also quite frequent (21%-16%), as well as the hypertensive (22%-18%) [34]. The mean age was 51 years. Also, in the InCor-HCFMUSP HF and Transplant Outpatient Clinic the etiology was ischemic in 28.2%, idiopathic in 28.2%, hypertensive in 20.6%, chagasic in 8.6%, tachycardiomyopathy in 2.1%, valvular in 6.5%, alcoholic in 2.1%, and peripartum in 3.2% [35]. At this clinic, comorbidities were found in significant proportions of patients: diabetes mellitus was diagnosed in 20.8%, chronic renal failure in 15.6%, dyslipidemia in 28.9%, hypothyroidism in 9.3%, and hyperuricemia in 5.2%. 

In other smaller randomized trials, the mean age of HF patients were 62-63 years, the etiology was ischemic in 25-35%, the hypertensive in 25-33% and others in 32-48% without Chagas disease because the study was developed in a non-endemic area [36]. 

Community Latin-American HF studies are scarce. In a population sample of 170 HF patients included by a family health program in the city of Niterói (Brazil), the mean age was 61 years, there was a female predominance (58%), and the etiology was hypertensive in 84%, and ischemic in 21% [37]. HF with preserved systolic function was observed in 64.2%. Smoking, coronary artery disease, diabetes mellitus, and chronic renal failure were more prevalent in systolic HF. The similar prevalence, 86.1% in HF with preserved systolic function versus 86.4 %, suggests that the systemic hypertension may have impact as a risk factor in both types of HF. 

In rural areas in Brazil, in a limited population, HF with preserved left ventricular ejection fraction diagnosed in 49% of patients was prevalent among women and in the presence of metabolic syndrome, while systolic HF was associated with males and ischemic etiology [38]. 

## PROGNOSIS OF HEART FAILURE

Prospective data about prognosis of HF patients in Latin America can be obtained only from clinical trials. Before the β-blockers were prescribed for HF, the GESICA trial reported 24-42% mortality during 13 months follow-up of patients with advanced HF [23]. In contrast, during the β-blocker era the Argentinean DIAL Trial reported 15-16% mortality during a mean 16 months follow-up. More recently in the β-blocker era the Brazilian REMADHE trial reported 36-43% mortality during a 2.47±1.75 years follow-up [25]. Thus, in the β-blocker era, mortality rates observed in Latin America trials are similar to those reported in the recently published ACCLAIM trial [39]. Moreover, assessment of the clinical status of Brazilian HF outpatients in the InCor-HCFMUSP HF Clinics according to the New York Heart Association functional class showed 32.3% in class I, 42.3 % in II, and 25.4 % in III [26].

Comorbidities may have impact in the rate of events in HF [40]. In Brazil, anemia was reported in 21% of outpatients in a community-based cohort, and in 25% of HF Clinic patients associated with worse renal function [41]. In patients with valvulopahty and HF, the prevalence of anemia, renal dysfunction, and some degree of malnutrition was 71.1%, 48.1%, and 25% respectively [42]. Renal failure was predictor of cardiac events independently of left ventricular ejection fraction [43].

## MANAGEMENT OF HEART FAILURE

In South America no community-based studies about management of HF have been published. The DIAL study on outpatients from Argentina showed use of diuretic in 82.5%, digoxin in 47%, amiodarone in 29.1%, spironolactone in 32.3%, angiotensin converting enzyme inhibitor in 79.6%, angiotensin receptor blocker in 13.4% and beta-blocker in 61.8% of HF patients [44]. Brazilian data from the REMADHE trial showed prescription of amiodarone in 9.65%, amlodipine in 5.4%, angiotensin receptor blocker in 14.5%, oral anticoagulant in 13.7%, angiotensin converting enzyme inhibitor in 82.5%, beta-blocker in 66%, spironolactone in 55%, hydralazine in 6.4%, nitrates in 11.7%, digoxin in 63.1%, diuretics in 81.7%, statin in 12.9%, resynchronization in 3.4%, and cardioverter-defibrillator in 0.64% [25]. Also, data from the HF and Transplant Outpatient Clinic of the Heart Institute of the University of São Paulo School of Medicine reported that most patients were receiving doses of medication close to ideal, according to the main established guidelines [26]. RAAS inhibitors were prescribed to 94.7% of patients whereas the remainder received the hydralazine-nitrate combination due to renal failure. A total of 84.7% received beta-blocker therapy; of those who were not using this class of drugs, 5.2% had stopped due to clinical signs of intolerance to the drug (dizziness and hypotension), whereas the remainder presented clear contraindications to its use (bronchospasm and severe peripheral artery disease). 

Overall, treatment of HF patients in South America is not uniform. In comparison with data from recent published ACCLAIM trial (Canada, Denmark, Germany, Israel, Norway, Poland, and USA) the use of beta-blocker, lipid-lowering drugs, antiplatelet, anticoagulants, implanted cardioverter defibrillator, and cardiac resynchronization is lower in South America [26]. In addition, in patients hospitalized for treatment of decompensated HF the BELIEF study in Brazil reported a lower rate use of beta-blockers, nitrates, angiotensin receptor blockers, angiotensin converting enzyme inhibitor, and a higher use of spironolactone [19]. Several factors are likely to explain these discrepancies between South America and USA or Europe including more limited resources, less adherence to guidelines, and differences regarding etiologies, and racial factors.

## FREQUENT AND NEGLECTED CAUSES OF HEART FAILURE IN SOUTH AMERICA

### Valvular Heart Disease

Rheumatic fever (RF) is the leading cause of valvular heart disease in Brazil, and is still widely prevalent [6, 45]. It is currently responsible for significant morbidity and mortality, being the cause of 90% of the cardiac surgeries in children and over 30% of the cardiac surgeries in adults, most of them of young age. Data from the Brazilian government health system show that RF and rheumatic heart disease (RHD) are still a major health problem, even in the 21st century [6]. There are annually 2200 hospital admissions for acute RF and it is possible to estimate that yearly 73.000 patients have acute RF in Brazil. If we accept that 30% of the patients with acute RF will develop valvular sequelae, we end up with 21900 new cases of RHD yearly. The increased risk of progressing to severe chronic valvar disease was associated with moderate or severe carditis, recurrences of acute RF, and mother´s low educational level [46]. In developed countries it has been possible to halt progression of RHD to more severe valvular heart lesions, if subclinical carditis is diagnosed early in the course of the disease and treated with secondary prophylaxis [47]**.** Unfortunately this is not the case in Brazil, because patients remain asymptomatic for 10 to 20 years, and only seek medical attention after presenting symptoms of HF secondary to RHD. 

Reliable data on the incidence of RF are scarce, especially in South America. There have been reports ranging from 7.9 per 100 habitants in La Paz (Bolivia) to 2.9 per 1000 habitants in Cuba [36, 48]. In 2003 101,822 children between 5 -14 years were reported with RHD from Latin America [49].

Currently, in Brazil and in most of Latin America, there is a trend toward decremental incidence rates of RF and RHD. However, while, most of the healthcare resources are spent in paying for high-complexity cardiac surgeries, little or no money has been directed toward prevention of the disease. There are presently in Brazil no specific programs for the prevention or early diagnosis of RF. Moreover, most physicians outside major centers are not sufficiently trained and have no access to diagnostic tests (such as echocardiography). These factors explain that many patients will seek medical attention at major tertiary centers when already having severe RHD. As a consequence, most such patients would require cardiac surgery. 

In a retrospective study involving seven pediatric rheumatology centers in the Sao Paulo State, Brazil, from January 1989 to December 1994, of 786 children and adolescents diagnosed with RF 404 were boys and 382 were girls, the mean age being 9.4 years [30]. Arthritis was present in 453 (57.6%) patients, carditis in 396(50.4%) and chorea in 274(34.8%). Erythema marginatum and subcutaneous nodules were observed in only 13 (1.65%) and 12 (1.5%) patients, respectively. Typical migratory poliarthritis occurred in 290 (64%) patients. Considering the 54% children with a diagnosis of rheumatic carditis, the valvular lesion most frequently observed was mitral insufficiency (75%), followed by aortic insufficiency (25%), tricuspid insufficiency (9%) and aortic stenosis (0.1%). Silent carditis and recurrences were common findings. In other report the rheumatic carditis was the most common manifestation of rheumatic fever, and low compliance with antibiotic contributed to recurrence and cardiac sequelae [50].

## CARDIOMYOPATHIES 

Reliable data about prevalence and incidence of cardiomyopathies in South America are lacking. Data limited to year 2006 about causes of death in the São Paulo State (Brazil) showed that cardiomyopathy is an important cause of death (Table **1**). There are no community studies. In general etiology data are obtained from hospitalized HF patients, patients under tertiary care or those included in clinical trials. Chagas heart disease and idiopathic cardiomyopathy are common diagnoses especially in some countries. Chronic Chagas´ disease was responsible for 8% of deaths (Table **1**). Alcoholic cardiomyopathy, hypertrophic cardiomyopathy and endomyocardial fibrosis are also reported, however their prevalences are low in comparison with other etiologies of HF [51]. Other restrictive and infiltrative cardiomyopathies are even more rarely found in reported series [52]. According the SEADE data from the São Paulo State (Brazil) the restrictive and pericardial etiologies (endomiocardial fibrosis, Loffler´s endocarditis, endocardial fibroelastosis, amyloidosis, pericardial diseases) were responsible by 0.41% of total HF deaths during 2006 (Table **1**).

In South America endomyocardial fibrosis is the most frequent cause of restrictive cardiomyopathies [53]. A case series of patients with endomyocardial fibrosis has been published from Brazil [54]. Angiographic data from patients with endomyocardial fibrosis show that 59% of them had biventricular involvement [55]. Tricuspid regurgitation was observed in 58% of these patients and mitral regurgitation in 60%. Analysis of factors influencing the course of endomyocardial fibrosis showed that biventricular involvement (moderate or severe), right ventricular fibrosis, ascites, atrial fibrillation, and presence of tricuspid and mitral regurgitation were associated with greater mortality [42, 56]. The presence of atrial fibrillation was associated with a greater prevalence of tricuspid regurgitation and fibrosis of right ventricle. In Venezuela the most frequent clinical feature of endomyocardial disease was HF associated with mitral regurgitation [57]. The mortality of patients with endomyocardial fibrosis in New York Heart Association functional class I/II and III/IV was 10% and 48% at five years follow-up [58]. Surgical excision of endocardial fibrous tissue has been performed early in the clinical course of endomyocardial fibrosis, thus allowing the preservation of the AV valves [59]. Successful heart transplantation was reported for treatment of end-stage HF due to endomyocardial fibrosis [60] (Table **2**).

## CHAGAS ´HEART DISEASE 

The most common feature of Chagas disease is chronic Chagas cardiomyopathy, which can present as conduction system abnormalities, brady- and tachyarrhythmias, biventricular dilated cardiomyopathy, apical aneurysm, and thrombus formation in the aneurysm or remodeled ventricles [61, 62]. Despite of higher incidence of thrombus formation, there is no evidence of greater pro-thrombotic status among patients with Chagas disease [63]. However, encephalic infarction was reported in 17.5% in autopsies of chagasic patients and it was accepted that its complications were associated with 52% 0f death of studied patients [64]. The clinical main manifestations of Chagas disease are HF, arrhythmias, sudden death, and embolism. In endemic regions or in cardiac centers receiving patients from endemic regions, Chagas' cardiomyopathy is the cause of heart failure in 8.1–41% of reported cases of HF in endemic countries [65-66]. In an estimated population of over 41 million, chronic Chagas heart disease was responsible for 8% of deaths caused by heart failure or diseases associated with heart failure. A tendency towards reduction of mortality rate due to Chagas disease has been observed in São Paulo State, the most populous state in Brazil from 1985 to 2006 [67]. However, chagasic HF is no longer limited to endemic countries [68].

Although the pathogenesis of chronic Chagas disease is not completely understood, the etiology of chagasic heart disease is likely multifactorial [69, 70]. Parasite persistence, inflammatory response, autoimmunity, damage to the parasympathetic system causing sympathetic over activity, and microvascular abnormalities, have all been studied extensively as possible pathogenic mechanisms. Most researchers in the field now agree that chronic low-grade parasite persistence drives tissue damage and the autoimmune component of Chagas cardiomyopathy. In fact, reactivation of Chagas infection after heart transplantation has reinforced evidence for a potential role of parasite persistence in the development of Chagas heart disease [71]. In general, histology of cardiac tissue from patients with chronic Chagas cardiomyopathy, combined with non-invasive investigations provide evidence of persistent myocarditis, inflammatory infiltrate oedema, contraction-band necrosis and myocytolysis, focal and diffuse areas of myocellular hypertrophy, and fibrosis that can affect the conduction system [72]. Co-infection has recently been reported in Chagas cardiomyopathy, but its impact on outcome remains unknown [73].

The prognosis of patients with heart failure due to Chagas´heart disease is worse in comparison with other etiologies [74, 75]. Recommendations of Guidelines for Treatment of Heart Failure due to Chagas´heart disease in general is similar to other etiologies, but with low degree of evidences [76, 77]. There is no evidence for specific agent treatment for chagasic heart failure [78]. Heart transplantation is effective in HF due to Chagas´heart disease but is associated to reactivation of the *T. cruzi* infection [79-80]. Alternative surgical procedures to heart transplantation have shown disappointing long-term results despite early benefits [81, 82]. Ventricular assist device followed by heart transplantation with success was reported for HF treatment due to Chagas heart disease [83, 84]. However, challenges in the management of chronic Chagas cardiomyopathy have not been properly addressed and specific trials for chronic Chagas cardiomyopathy are now both welcome and necessary.

## CLINICAL IMPLICATIONS

Epidemiologic data are pointing to an increment in HF incidence in the future in South America as a consequence of factors of epidemiologic transition, advances in health care, aging of the population, and prevalence of coronary artery disease, cigarette smoking, hypertension, diabetes and obesity [85]. Favorable trends for reduction in risk factors leading to reduction in deaths due to cardiovascular disease in developed countries should be a model for South America Countries [86]. Unfortunately, data show a increment in hypertensive causes of death. Also, the conduction of epidemiologic HF studies is an urgent need to guide the implementation of preventive interventions and appropriate treatments, because of limited available resources. If timely preventive and treatment interventions are not introduced, HF could become one of the main contributors to the burden of morbidity, mortality, and health costs in South America [55]. Even in recent years, the public health priorities in developing countries [87], which are set by government politics makers in these countries and by international agencies, have been the reduction of infant and maternal mortality and to control infectious disease. The results of the present study call for serious attention to be given to the consequences of chronic diseases such as HF in developing countries [88].

There is a lack of good quality prevalence surveys of rheumatic heart disease in South America [31, 89]. Data suggest that rheumatic fever remains as an unsolved problem of public health [90]. The persistent high morbidity and mortality due to rheumatic heart disease in Brazil, with high-cost procedures to treat valvular sequelae, warrant procedures to improve primordial prevention (housing, hygiene), primary prevention-prophylaxis (sore-throat treatment), secondary prevention-prophylaxis, early diagnosis of asymptomatic rheumatic carditis, and tertiary prevention (medication for HF, valve surgery, anticoagulation) [36]. Also, it should stimulate research addressing factors associated with rheumatic heart disease such as the organism (group A streptococci), genetic, host and environmental factors (socio-economic status, living conditions, overcrowding, urbanization, daycare [32], nutrition and access to medical services). The World Health Organization has recommended secondary prophylaxis, most effectively delivered within a coordinated program using a registry of patients [91], however, most developing countries still do not have a secondary-prophylaxis program. The beneficial effects of a program for prevention and control of rheumatic fever and rheumatic heart disease were published recently [92].

The morbidity and mortality secondary to Chagas´heart disease necessitate the development and maintenance of programs to control the vector, eliminated transmission, and clinical trials to improve the HF treatment and prevention of sudden death. (Table **1**) Also, clinical trials to evaluate the neglected specific treatment to prevent the Chagas´heart disease or its worsening are highly desirable [93].

## CONCLUSION

Results from this review indicate that cardiovascular disease is the predominant cause of death. Also, transition from pandemics of infection to predominant degenerative chronic disease is taking place in most South America developing countries. HF is likely become a major public burden, but the exact magnitude of problem related to HF in South America is unknown and the causes of HF may vary widely among countries. RHD and Chagas’ heart disease are still remaining as common causes of HF in many countries of South America. The prevention of rheumatic heart disease and HF is an important public health and it should deserve high priority.

## Figures and Tables

**Table 1.** Causes of Deaths due to HF or Etiologies Associated with HF Based on the SEADE Data from São Paulo State (Brazil) during 2006 According International Classification of Diseases (ICD)-10. Total deaths: 242,832 in estimated 41 654,020 habitants

ICD Code	Cause	Number	% of HF Death	% of Total Death
B57	Chagas´disease	1197	7.81	0.49
B57.0+	Acute with Heart Involvement	1	0.007	0.0004
B57.2	Chronic with Heart Involvement	1196	7.8	0.49
E63.9	Nutritional Cardiomyopathy	1	0.007	0.0004
E85	Amyloidosis	23	0.15	0.0095
E85.2	Heredofamilial, unspecified	1	0.007	0.0004
E85.3	Secondary systemic	4	0.026	0.0016
E85.4	Organ-limited amyloidosis	5	0.033	0.0021
E85.8	Other amyloidosis	2	0.013	0.0008
E85.9	Amyloidosis, unspecified	11	0.072	0.0045
I10	Hypertensive (Hip) Diseases	2128	13.88	0.88
I11.0	Hip.Heart disease with HF	1831	11.94	0.75
I13.0	Hip.Heart Disease with Renal Disease and HF	6	0.039	0.0025
I13.1	Hip.Heart and Renal Failure	101	0.66	0.042
I13.2	Hip.Heart and Renal disease with Renal Failure and HF	190	1.24	0.078
I25.5	Ischemic Cardiomyopathy	1404	9.16	0.58
I31.1	Chronic Constrictive Pericarditis	7	0.046	
I42	Cardiomyopathy	3571	23.2	1.47
I42.0	Dilated Cardiomyopathy	2638	17.2	1.09
I42.3	Endomyocardial Disease (EMF, Löffler´s endocarditis)	6	0.039	0.0025
I42.4	Endocardial Fibroelastose	24	0.16	0.0099
I42.5	Other Restrictive Cardiomyopathy	3	0.02	0.0012
I42.6	Alcoholic Cardiomyopathy	69	0.45	0.028
I42.7	Drugs and other External Agents	2	0.013	0.0008
I42.8	Other Cardiomyopathies	8	0.052	0.0033
I42.9	Cardiomyopathy, unspecified	821	5.3	0.34
I50	HF	6468	42.18	2.66
I50.0	Congestive Heart Failure	3593	23.43	1.48
I50.1	Left Ventricular Failure	564	3.68	0.23
I50.9	HF, unspecified	2311	15.07	0.95
I51.7	Cardiomegaly	458	2.99	0.19
I57.0	Cardiogenic Shock	79	0.52	0.03
	Total	15336		6.3%

HF means Heart Failure; EMF, endomyocardial fibrosis; SEADE means SEADE Foundation -(Fundação Sistema Estadual de Análise de Dados).

**Table 2.** HF Data and Studies from South America

Source	Deaths(d) or Patients (pts)	Mean Age	Male in %	Etiology in %	Mortality due to HF
**General**					
SEADE	242,832 deaths	-----	----	Isch 9, Ch 8, Hyp 14, CMP 23, HF 42, Amyloidosis 0.15	6.3% of total death
Inpatients					
DATASUS	39% of 743,763 hospitalized pts	----	----	---------------	6.6-7%
Godoy HL	194 098 pts				15%
Barreto AC	903 pts	53	60	Isch 34, IDC 26, Val 22, Hyp 7, Ch 6	-----
Perna ER	2974 pts	65-70	55-66	Isch 27-38, Ch 1.3-84, hyp 18-32, Val 16-22, IDC 1.3-14, Alco 0-5.4	12.1-4.7%
Tavares LR	203 pts	61-72	50-49	Isch 62-65	5.2-2.94%
Rohde LE	143 pts	73	50	Isch 39, Hyp 25, Val 10, other 26	13%
Mangini S	212 pts	60	56	Isch 30, Hyp 21, Val 15, Ch 15, IDC 8, other 12	10%
Bocchi EA (BELIEF Study)	182 pts	55	67	Isch 34, Ch 21, Hyp 13, IDC 31	14.8%
Outpatients					
Freitas HFG	1220 pts	46	78	IDC 37, Ch 20, Isch 17, hyp 14, others 12	34% at 26m
GESICA trial	516 pts	60-59	82-79	Isch 38-40, IDC 20-23, Ch 11-8	33-26% at 12m
Mendez GF	45 pts	61	53	Isch 47, IDC 44	12% at 6m
Bocchi EA (REMADHE trial)	350 pts	52-50	64-71	Isch 22-28,Alco 8-4, Ch 21-16, Hyp 22-18, IDC 10-17, Val 3, other 14	25-46% at 36m
Silva CP	96 pts	52	60	Isch 28, IDC 28, Hyp 21, Ch 9, Tachycar 2.1, Val 7, Alco 2, Peripartum 5	-----
DIAL trial	1518 pts	65	73-69	Isch 43-45	12.5% at 12m
Domingues FB	120 pts	63-62	51-68	Isch 27-35%, hyp 25-33%	17% at 3 m
Community					
Moutinho	170 pts	61	42	LVEF nl 64% of pts	-----

General means general population; SEADE, SEADE Foundation of the State System of Data Analysis(Fundação Sistema Estadual de Análise de Dados); DATASUS, Brazilian Ministry of Health data; Isch, ischemic etiology; Ch, chagasic etiology; Hyp, hypertensive; CMP, cardiomyopathy; HF, heart failure; Val, valvular etiology; Alco, alcoholic; IDC, idiopathic dilated cardiomyopathy; tachycard; tachycardiomyopathy; pts, patients; LVEF, left ventricular ejection fraction; nl, normal; m, months
